# An in vivo screen identifies NAT10 as a master regulator of brain metastasis

**DOI:** 10.1126/sciadv.ads6021

**Published:** 2025-03-26

**Authors:** Jocelyn F. Chen, Peng Xu, Wesley L. Cai, Huacui Chen, Emily Wingrove, Xiaojian Shi, Wenxue Li, Giulia Biancon, Meiling Zhang, Amer Balabaki, Ethan D. Krop, Elianna Asare, Yangyi Zhang, Mingzhu Yin, Toma Tebaldi, Jordan L. Meier, Thomas F. Westbrook, Stephanie Halene, Yansheng Liu, Hongying Shen, Don X. Nguyen, Qin Yan

**Affiliations:** ^1^ Department of Pathology, Yale School of Medicine, New Haven, CT 06520, USA.; ^2^ Department of Cellular and Molecular Physiology, Yale School of Medicine, New Haven, CT 06510, USA.; ^3^ Systems Biology Institute, Yale West Campus, West Haven, CT 06516, USA.; ^4^ Department of Pharmacology, Yale Cancer Biology Institute, Yale University, West Haven, CT 06516, USA.; ^5^ Yale Cancer Center, Yale School of Medicine, New Haven, CT 06520, USA.; ^6^ Department of Internal Medicine (Section of Hematology), Yale School of Medicine, New Haven, CT 06520, USA.; ^7^ Department of Cellular, Computational and Integrative Biology (CIBIO), University of Trento, Trento, Italy.; ^8^ Chemical Biology Laboratory, National Cancer Institute, National Institutes of Health, Frederick, MD 21702, USA.; ^9^ Therapeutic Innovation Center (THINC), Verna & Marrs McLean Department of Biochemistry & Molecular Pharmacology, Department of Molecular and Human Genetics, and Dan L Duncan Comprehensive Cancer Center, Baylor College of Medicine, Houston, TX 77030, USA.; ^10^ Yale Stem Cell Center, Yale School of Medicine, New Haven, CT 06520, USA.; ^11^ Department of Biomedical Informatics & Data Science, Yale School of Medicine, New Haven, CT 06510, USA.; ^12^ Department of Internal Medicine (Section of Medical Oncology), Yale School of Medicine, New Haven, CT 06520, USA.; ^13^ Yale Center for Immuno-Oncology, Yale School of Medicine, New Haven, CT 06520, USA.; ^14^ Yale Center for Research on Aging, Yale School of Medicine, New Haven, CT 06520, USA.

## Abstract

Emerging evidence has shown that epigenetic regulation plays a fundamental role in cancer metastasis, the major cause of cancer-related deaths. Here, we conducted an in vivo screen for vulnerabilities of brain metastasis and identified *N*-acetyltransferase 10 (NAT10) as a driver of brain metastasis. Knockdown of NAT10 restrains cancer cell proliferation and migration in vitro and tumor growth and brain metastasis in vivo. The poorly characterized RNA helicase domain of NAT10 is critical for cell growth in vitro, while both RNA helicase and NAT domains are essential for primary tumor growth and brain metastasis in vivo. Mechanically, NAT10 promotes the expression of 3-phosphoglycerate dehydrogenase (PHGDH) and phosphoserine aminotransferase 1 (PSAT1), two enzymes for serine biosynthesis implicated in brain metastasis. Silencing *PHGDH* or *PSAT1* in metastatic breast cancer cells inhibits their growth in the serine/glycine-limited condition, phenocopying the effects of NAT10 depletion. These findings establish NAT10 as a key regulator of brain metastasis and nominate NAT10 as a target for treating metastasis.

## Teaser

NAT10 is identified as a master regulator of brain metastasis and a potential target for treating metastasis.

## INTRODUCTION

Metastasis is responsible for 90% of cancer-associated mortality (*1*). Brain metastases, found in 15 to 35% of patients with breast cancer and 16 to 27% of patients with lung cancer (*2*, *3*), are associated with the worst outcome compared to metastasis to other organs (*4*). Treatment options for these patients, especially those with triple-negative breast cancer (TNBC) brain metastasis, are limited. The median survival from the diagnosis of TNBC central nervous system metastases is only 4.9 months (*5*, *6*). Thus, effective approaches to treating brain metastasis are urgently needed.

Epigenetic regulation has been found in all the steps of metastasis cascades (*1*). Studies from our group and others have shown the profound effects of epigenetic alterations on the expression of cancer-essential genes or metastasis-promoting genes, which subsequentially modulate cancer cell proliferation, metastatic capability, and the adaptation to the distal metastatic sites (*7*–*9*). However, the epigenetic dependencies of brain metastasis are largely unexplored.

Here, we constructed screens to specifically investigate the epigenetic dependencies of breast cancer brain metastasis (BCBM). We established *N*-acetyltransferase 10 (NAT10) as a driver of BCBM with extensive evidence from in vitro experiments and preclinical studies. NAT10 is the only identified NAT that “writes” *N*^4^-acetylcytidine (ac4C) on RNA in mammals. It acetylates multiple species of RNA, including ribosomal RNA (rRNA), tRNA, and possibly mRNA, and also appears to carry out lysine acetylation on proteins including histones (*10*–*14*). Emerging studies have revealed the role of NAT10 in tumorigenesis and metastasis via its NAT function (*15*–*18*). NAT10 also harbors an RNA helicase domain, the function of which is obscure in cancer biology. Here, we show that, mechanistically, NAT10 regulates 3-phosphoglycerate dehydrogenase (PHGDH) and phosphoserine aminotransferase 1 (PSAT1) via its RNA helicase domain to support the proliferation of metastatic breast cancer cells in the serine/glycine-limited brain microenvironment.

## RESULTS

### An in vivo screen identifies NAT10 as a top dropout hit required for BCBM

To facilitate brain metastasis screen, we performed in vivo selection for brain metastatic derivatives of the MDA-MB-231-BrM2 (231-BrM2), a brain metastasis derivative of MDA-MB-231 TNBC cells (*19*), and generated the MDA-MB-231-BrM3 (231-BrM3) derivative line with higher brain metastasis potential (fig. S1A). Compared with 231-BrM2, 231-BrM3 showed significantly increased brain metastasis ability as measured by bioluminescence in vivo and ex vivo (fig. S1, B and C), making it an ideal tool to screen for regulators of brain metastasis.

**Fig. 1.**
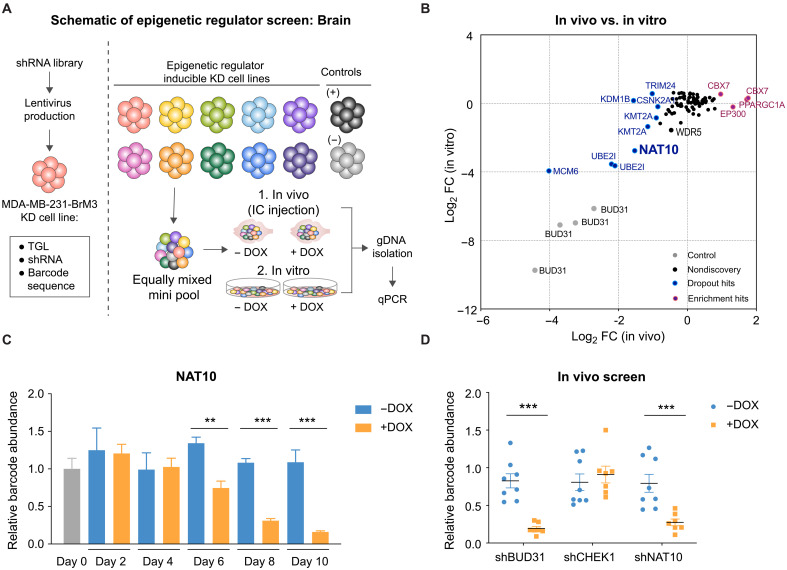
In vivo and in vitro screens identified epigenetic dependencies of BCBM. (**A**) Schematic of shRNA screens for identifying the epigenetic dependencies of BCBM. pGIPZ plasmid harboring barcode and hairpin targeting certain epigenetic factor was digested and subcloned into the pINDUCER10 plasmid, which contains HSV1-TK/GFP/Fluc (herpes simplex virus 1–thymidine kinase/green fluorescent protein/firefly luciferase) (TGL) triple reporter gene. Epigenetic regulator inducible knockdown (KD) cell lines were equally mixed and injected into mice or cultured under control or DOX (1 μg/ml)–treated condition. Brain (in vivo) and cells (in vitro) were harvested for gDNA and subjected to barcode qPCR as the screening output. IC, intracardiac. (**B**) Log_2_ (fold change) of the in vivo versus in vitro screen results for each epigenetic regulator. FC, fold change. (**C**) Relative abundance of barcode for shRNA against *NAT10* in cells cultured with control or DOX treatment. (**D**) Relative abundance of barcode for shRNA against *NAT10*, *BUD31*, and *Checkpoint kinase 1* (*CHEK1*) in the brain tissue from control and DOX-treated mice. *BUD31* and *CHEK1* serve as positive and negative controls, respectively. Significance in (C) and (D) was determined using unpaired Student’s *t* test. **P < 0.01; ***P < 0.001.

To identify epigenetic dependencies for BCBM, we conducted parallel in vivo and in vitro functional screens using an inducible, barcoded short hairpin RNA (shRNA) library (Fig. 1A). This shRNA screening strategy was successfully used in our previous breast cancer lung metastasis study (*7*). Briefly, we compiled a list of epigenetic regulators based on the availability of small-molecule inhibitors and the association between their expression and poor survival. We tested the knockdown efficiency of 326 shRNAs targeting 89 actionable epigenetic regulators and subcloned one shRNA with the best knockdown efficiency per target gene into the doxycycline (DOX) inducible and barcoded pINDUCER10 lentivirus backbone (*7*). shRNA against *BUD31*, which was previously shown to be essential for cell proliferation (*20*), was included as the positive control, while shRNA against *CHEK1* was included as the negative control (*7*). To enhance the representation of each factor in the in vivo screen, we generated six minipools by equally mixing 11 to 13 individual inducible epigenetic regulator knockdown BrM3 cell lines with the positive and negative control cell lines mentioned above. We injected minipools (5 × 10^5^ cells) intracardially into mice for in vivo screen (Fig. 1A) and monitored brain metastases weekly with in vivo live imaging. At the end point (5 weeks), we harvested the brains for genomic DNA (gDNA) isolation and quantitative polymerase chain reaction (qPCR) quantification. For the in vitro screen, we pooled the cell lines in equal numbers, cultured them under either control or DOX conditions for up to 10 doublings, and harvested them every 2 days for gDNA extraction and qPCR quantification (Fig. 1A).

Of the 69 epigenetic regulators that were targeted in this screen, we identified 10 significant and consistent hits [*P* < 0.05, log_2_ (fold change) > 0.8 or <−0.8], including 7 dropout and 3 enrichment hits (Fig. 1B and fig. S2, A to G), with positive and negative controls showing corresponding phenotypes (fig. S2, H and I). Some in vivo dropout candidates also exhibited dropout phenotype in vitro, including *MCM6*, *UBE2I*, and *NAT10* (Fig. 1, B to D). MCM6 is an essential factor for DNA replication initiation (*21*), and it was reported that MCM6 repression inhibits TNBC lung metastasis (*22*). UBE2I/UBC9, the sole known E2 ubiquitin–conjugating enzyme, was shown to promote growth of breast cancer in mice (*23*). NAT10, the only ac4C NAT in mammals, acetylates multiple RNA species and proteins to modulate their stability and activity (*12*, *13*). However, the roles of NAT10 in brain metastasis have not been investigated.

### Silencing of *NAT10* inhibits breast cancer cell proliferation

**Fig. 2.**
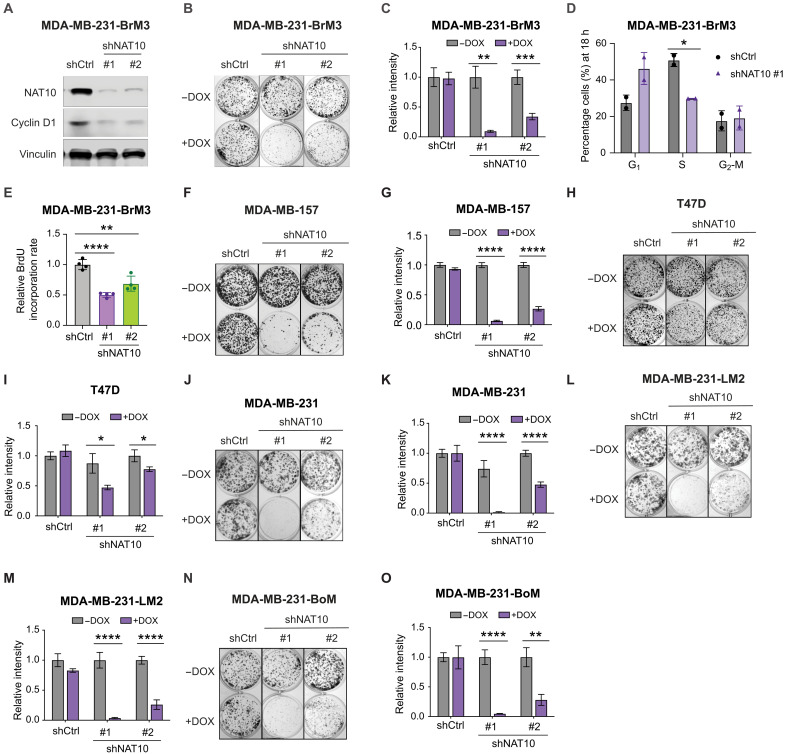
NAT10 promotes breast cancer proliferation across subtypes and metastatic organotropisms. (**A**) Western blot of indicated proteins in 231-BrM3 cells harboring inducible control or *NAT10* targeting shRNAs (shNAT10 #1 and shNAT10 #2) after 3 days of DOX (1 μg/ml) induction. (**B** and **C**) Colony formation assays of 231-BrM3 cells with *NAT10* knockdown or control after 9 days of either control or DOX (1 μg/ml) treatment. Representative images (B) and quantification (C) are shown. (**D**) Percentage of cells in G_1_, S, and G_2_-M phase from indicated cells fixed at 18 hours post-refeeding. (**E**) BrdU incorporation assay of 231-BrM3 cells after 3 days of DOX (1 μg/ml) induction. (**F** to **O**) Colony formation assays of indicated cells after 9 days of either control or DOX (1 μg/ml) treatment. Representative images [(F), (H), (J), (L), and (N)] and quantification [(G), (I), (K), (M), and (O)] are shown. Significance in (C) to (E), (G), (I), (K), (M), and (O) was determined using unpaired Student’s *t* test. *P < 0.05; **P < 0.01; ***P < 0.001; ****P < 0.0001.

To dissect the functions of NAT10 in breast cancer, we generated two independent *NAT10* inducible knockdown 231-BrM3 cell lines (Fig. 2A) and demonstrated that knockdown of *NAT10* significantly decreased the clonogenic ability of 231-BrM3 (Fig. 2, B and C). Reduced cell proliferation can occur secondary to the disruption of cell cycle. To identify which phase within cell cycle progression is affected, we synchronized the cells at the G_2_-M phase with nocodazole and monitored the percentage of cells entering each phase of cell cycle after release from G_2_-M arrest (fig. S3A). *NAT10* knockdown cells exhibited slower progression from the G_2_-M to the S phase (Fig. 2D and fig. S3B). Consistently, *NAT10* knockdown cells showed a decreased 5-bromo-2′-deoxyuridine (BrdU) incorporation rate compared to control cells (Fig. 2E), indicating that NAT10 is critical for cells to enter the S phase. Knockdown of *NAT10* down-regulated cyclin D1 (Fig. 2A), a key regulator of the cell cycle progression. Reduced clonogenic ability could also be due to enhanced cell death; therefore, we assessed whether *NAT10* knockdown results in apoptosis in 231-BrM3 cells. Only less than 1% of cells in *NAT10* knockdown and control groups undergo apoptosis (fig. S3, C and D), suggesting that knockdown of *NAT10* does not induce apoptosis.

### NAT10 promotes proliferation across breast cancer subtypes and metastatic organotropisms

We further asked whether NAT10-regulated cell proliferation is a general phenomenon in other breast cancer subtypes and MDA-MB-231 derivatives with distinct organotropism. We examined the effects of *NAT10* knockdown on clonogenic ability in an additional TNBC cell line MDA-MB-157 and an estrogen receptor (ER)–positive breast cancer cell line T47D. Knockdown of *NAT10* impaired the clonogenic ability of MDA-MB-157 and T47D (Fig. 2, F to I, and fig. S3, E and F). The effect of NAT10 loss on MDA-MB-157 and 231-BrM3 is stronger than that on T47D, suggesting that TNBC cells may be more dependent on NAT10 than ER-positive breast cancer cells.

To address whether NAT10 is essential for the growth of TNBC cells with distinct organotropism, we turned to two other organotropic derivatives of MDA-MB-231, MDA-MB-231-LM2 (231-LM2), and MDA-MB-231-BoM (231-BoM). Having been selected in vivo similarly to 231-BrM3, 231-LM2 and 231-BoM cells preferentially metastasize to the lung and bone, respectively (*19*, *24*). We depleted NAT10 with two independent shRNAs in parental MDA-MB-231, 231-LM2, and 231-BoM (fig. S3, G and H) and found that knockdown of *NAT10* significantly impaired cell proliferation and colony formation in parental MDA-MB-231, 231-LM2, and 231-BoM to similar degrees as in 231-BrM3 cells (Fig. 2, J to O, and fig. S3, I to K). These results demonstrate that NAT10 regulates cell proliferation independent of their metastatic organotropism.

### NAT10 is required for breast tumor growth and metastasis

**Fig. 3.**
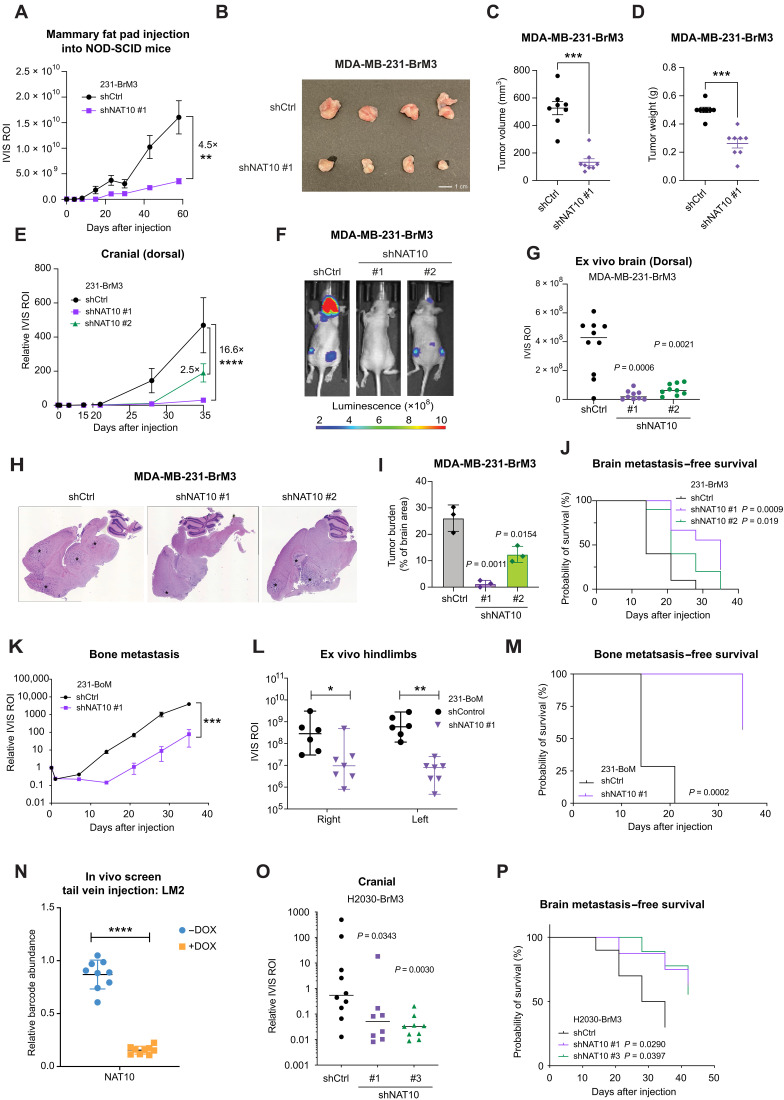
NAT10 is essential for primary breast tumor growth and brain metastasis. (**A**) Bioluminescence signals of mice injected into the fourth mammary fat pad with 231-BrM3 cells harboring inducible shCtrl or shNAT10 #1. ROI, region of interest. (**B**) Representative images of primary tumors from mice in (A) on day 58. (**C** and **D**) Quantification of primary tumor volume (C) and weight (D) from mice in (A) on day 58. (**E**) Normalized bioluminescence signals of brain metastases of mice. (**F**) Representative bioluminescence images of mice in (E) on day 28. (**G**) Quantification of ex vivo bioluminescence signals of the brain tissues from mice in (E) on day 35 postinjection. (**H**) Representative hematoxylin and eosin (H&E) stain of sagittal brain tissue slides. Brain metastases were marked with *. (**I**) Quantification of tumor burden. *n* = 3 for each group. (**J**) Kaplan-Meier plot of brain metastasis–free survival in (E). shCtrl (*n* = 10), shNAT10 #1 (*n* = 9), and shNAT10 #2 (*n* = 10). (**K**) Normalized bioluminescence signals of bone metastasis of mice. (**L**) Quantification of ex vivo bioluminescence signals of hindlimbs (left and right) from mice in (K) on day 35. (**M**) Kaplan-Meier plot of bone metastasis–free survival in (K). (**N**) Relative abundance of barcode for shRNA against *NAT10* in lung tissue from control and DOX-treated mice. (**O**) Quantification of brain metastasis burden from mice on day 35. (**P**) Kaplan-Meier plot of brain metastasis–free survival in (O). Unpaired two-tailed Student’s *t* tests were used in (A), (E), (I), (K), and (N) with the data representing average ± SEM. Unpaired Mann-Whitney tests were used in (C), (D), (G), (L), and (O), and each dot represents one mouse. Log-rank Mantel-Cox tests were used in (J), (M), and (P). *P < 0.05; **P < 0.01; ***P < 0.001; ****P < 0.0001.

To test whether knockdown of *NAT10* affects orthotopic tumor growth in vivo, we injected 231-BrM3 cells with or without *NAT10* knockdown to the fourth mammary fat pads in mice. A significant decrease in bioluminescence signal in mammary glands was observed in the *NAT10* knockdown group compared to the control group (Fig. 3A). At the end point (day 58), the tumor size and weight in the *NAT10* knockdown group were significantly reduced compared to the control tumors (Fig. 3, B to D).

To address whether NAT10 is required for BCBM, we injected 231-BrM3 cells with or without *NAT10* knockdown through the left ventricle of mice. We monitored brain metastatic colonization and outgrowth over 35 days and observed a significant impairment on brain colonization in the *NAT10* knockdown group (Fig. 3, E to G). At the end point (day 35), the average brain metastatic burden in mice with stronger *NAT10* knockdown efficacy (shRNA #1; Fig. 2A) was 16.6-fold lower than that in mice injected with 231-BrM3 control cells (Fig. 3, E and F). Notably, the difference in brain metastasis exceeds the difference in mammary tumor growth (comparing Fig. 3E with Fig. 3A), implying that NAT10 has brain metastasis–specific roles. Consistently, mice from the *NAT10* knockdown group showed decreased brain metastases burden as quantified via ex vivo brain luminescent signal at the end point (Fig. 3G) and as evidenced by the hematoxylin and eosin (H&E) staining of brain tissue sections (Fig. 3, H and I).

As a small proportion of 231-BrM3 cells can metastasize elsewhere in addition to the brain, we monitored whole-body metastasis and observed decreased overall metastatic burden in *NAT10* knockdown groups (fig. S4A). Mice injected with NAT10-depleted 231-BrM3 cells showed delayed development of brain metastases and extracranial metastases compared to the control mice (Fig. 3J and fig. S4B). These results suggest that NAT10 is a critical factor for the development of breast cancer metastasis to the brain and other organs.

### NAT10 is an essential factor for breast and lung cancer metastases to multiple organs

Because NAT10 depletion also impaired whole-body metastasis burden (fig. S4A) and knockdown of NAT10 in both 231-BoM and 231-LM2 led to significant decrease in cell growth (Fig. 2, L to O, and fig. S3, I and J), we asked whether NAT10 affects breast cancer metastasis to other organs such as the bone and lungs. We injected 231-BoM cells with or without *NAT10* knockdown intracardially and monitored bone metastasis colonization and outgrowth especially at the hindlimb area. We observed a significant impairment on bone colonization in the *NAT10* knockdown group (Fig. 3K). Consistently, mice from the *NAT10* knockdown group also showed decreased hindlimb metastasis burden in both sides from the quantification of ex vivo bone luminescent signal at the end point (Fig. 3L). Mice injected with NAT10-depleted 231-BoM cells showed delayed development of bone and nonbone metastases compared to the control mice (Fig. 3M and fig. S4C). In addition to bone metastasis, we revisited our previous screening results of breast cancer lung metastasis (*7*) and found that 231-LM2 cells with NAT10 ablation was significantly dropped out in the lungs (Fig. 3N).

Among the brain metastasis cases, metastases originating from lung cancer account for the highest percentage (*25*). To test whether NAT10 is required for brain metastasis by lung cancer, we used H2030-BrM3 cells, brain metastatic derivatives of the H2030 non–small cell lung cancer cell line harboring the *KRAS^G12C^* mutation. We depleted NAT10 using shRNA (fig. S4D) and injected H2030-BrM3 with or without *NAT10* knockdown intracardiacally into the mice. Knockdown of *NAT10* significantly decreased the brain metastasis ability of H2030-BrM3 (Fig. 3O). In addition, mice injected with NAT10-depleted H2030-BrM3 cells showed delayed development of brain metastases and extracranial metastases compared to the control mice (Fig. 3P and fig. S4E), suggesting that NAT10 is important for lung cancer brain and extracranial metastases. Together, our data indicate that NAT10 is a driver of breast cancer and lung cancer metastases to multiple organs.

### The RNA helicase function of NAT10 is critical for breast cancer cell growth and migration

**Fig. 4.**
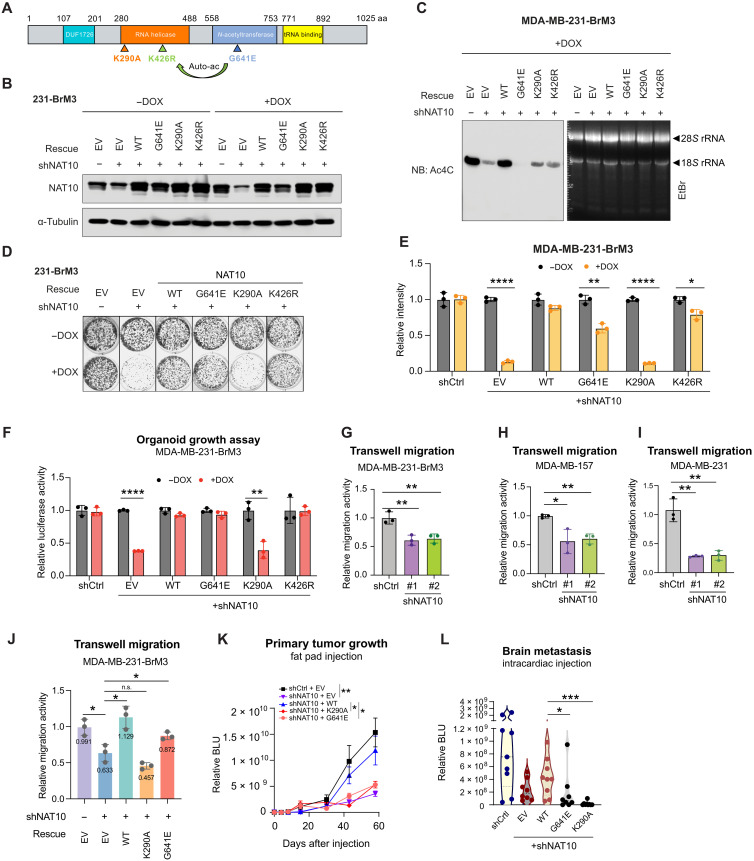
RNA helicase and NAT domains are required for NAT10-mediated phenotypes. (**A**) Schematic of NAT10 with indicated domains and mutation sites. G641E, acetyltransferase dead mutant; K290A, helicase dead mutant; K426R, NAT10 autoacetylation site mutant. aa, amino acids. (**B**) Western blot of indicated proteins in 231-BrM3 cells with inducible control or shNAT10, followed by rescuing with empty vector (EV), shRNA-resistant WT NAT10, or mutants. Cells were collected after 3 days of control or DOX (1 μg/ml) induction. (**C**) Immuno–Northern blots (NB) of ac4C modification in total RNA extracted from indicated 231-BrM3 cells. Cells were induced with DOX (1 μg/ml) for 5 days. (**D** and **E**) Colony formation assays of indicated 231-BrM3 cells after 9 days of either control or DOX (1 μg/ml) treatment. Representative images (D) and quantification (E) are shown. (**F**) Organoid growth assays of indicated 231-BrM3 cells after 12 days of either control or DOX (1 μg/ml) treatment. The three-dimensional (3D) growth of cells was determined by in vitro luciferase activity and normalized to control. (**G** to **I**) Transwell migration assays of 231-BrM3 (G), MDA-MB-157 (H), and MDA-MB-231 (I) cells after 3 days of DOX (1 μg/ml) induction. (**J**) Transwell migration assays of 231-BrM3 cells with indicated genetic manipulations after 5 days of DOX (1 μg/ml) induction. Mean values reflecting the relative migration abilities of each group are labeled in the plot. n.s., not significant. (**K**) Quantification of bioluminescence (BLU) signals of mice injected into the fourth mammary fat pad with indicated 231-BrM3 cells. The data represent average ± SEM. (**L**) Quantification of BLU signals of brain metastases in mice on day 35. Each dot represents one animal. Unpaired two-tailed Student’s *t* test was used in (E) to (K), while unpaired two-tailed Mann-Whitney test was used in (L). *P < 0.05; **P < 0.01; ***P < 0.001; ****P < 0.0001.

We next sought to examine which molecular function of NAT10 is required for breast cancer cell growth. To this end, we performed structural-functional analysis of NAT10 by constitutively expressing shRNA-resistant wild-type (WT) *NAT10* and three *NAT10* mutants (G641E, acetyltransferase dead; K290A, helicase dead; K426R, mutant of NAT10 autoacetylation site) (*26*–*28*) with N-terminal Flag-tag in the *NAT10* knockdown cell line (Fig. 4A). We optimized the condition for DOX induction so that the expression levels of ectopic NAT10 (WT or mutants) were comparable to endogenous NAT10 in the indicated cell lines, whereas endogenous NAT10 was significantly depleted (Fig. 4B).

As NAT10 is capable of acetylating several reported RNAs and proteins, we assessed whether such acetyltransferase activity is active in 231-BrM3 and whether such modification contributes to the observed growth phenotype. We identified a strong band at the position of 18*S* rRNA, a known RNA substrate of NAT10 (*11*), with ac4C antibody in immuno–Northern blot, while the signal decreased when NAT10 was inducibly depleted (fig. S5A). In the time course experiment, the ac4C on 18*S* rRNA decreased over time (fig. S5B). We also observed light NAT10-dependent smears occurring in the size range expected for poly(A) RNAs (fig. S5, A and B). Similarly, NAT10-dependent 18*S* rRNA acetylation was detected in H2030-BrM3 cells (fig. S5, C and D). We then examined the RNA acetylation in NAT10 rescued cell lines and observed that reexpressing WT NAT10 restored the ac4C modification on 18*S* rRNA (Fig. 4C). The 18*S* rRNA acetylation was completely abolished when the G641E mutant was introduced, indicating that the G641E mutant serves a dominant negative mutant, consistent with the notion that this residue is critical for NAT10’s acetyltransferase activity (*26*). The K290A and K426R mutants were unable to fully rescue 18*S* rRNA acetylation (Fig. 4C), indicating that these mutants also have impaired acetyltransferase activity. Together, these results confirmed that NAT10 is responsible for RNA ac4C modification in breast and lung cancer cells.

To determine which function of NAT10 contributes to cell growth, we performed colony formation assays using the rescued cell lines treated with DOX or dimethyl sulfoxide. We observed that reexpressing WT NAT10 rescued the growth phenotype by around 85%, a similar result was observed when we reexpressed the K426R mutant (Fig. 4, D and E). Unexpectedly, G641E mutant could partially rescue the growth to more than 50%. In contrast, K290A mutant could not rescue the growth at all (Fig. 4, D and E). To examine the roles of NAT10 in a more physiologically relevant setting, we performed a three-dimensional (3D) organoid growth assay by culturing 231-BrM3 cells in suspension with 5% Matrigel. Consistently, NAT10 K290A mutant reexpression did not rescue the 3D growth of 231-BrM3 cells while WT, G641E, and K426R mutants did (Fig. 4F). The ability of NAT10 to promote metastasis (Fig. 4 and fig. S4) prompted us to assess its roles in migration using transwell migration assays. We found that NAT10 loss decreased the migration of 231-BrM3, MDA-MB-157, and MDA-MB-231 cells (Fig. 4, G to I). Similarly, NAT10 K290A mutant did not rescue transwell migration of 231-BrM3 cells while WT and G641E mutant did (Fig. 4J). These results suggest that the RNA helicase function, but not the acetyltransferase activity or autoacetylation of NAT10, is most critical for breast cancer cell growth and migration.

### The NAT and RNA helicase functions of NAT10 are essential for breast cancer growth and brain metastasis in vivo

To further explore which function of NAT10 is essential for the growth of 231-BrM3 in vivo, we injected the same panel of genetically manipulated 231-BrM3 cells into the fourth mammary fat pad of nonobese diabetic–severe combined immunodeficient mice (NOD-SCID) mice to evaluate their effects on primary tumor growth. As expected, WT NAT10 was able to rescue the primary tumor growth (Fig. 4K). However, neither the G641E mutant nor the K290A mutant was able to rescue tumor growth (Fig. 4K). When injected intracardiacally into the mice, NAT10 K290A and G641E mutant–rescued cells also exhibited impaired brain metastasis colonization and outgrowth as compared to the WT NAT10–rescued cells (Fig. 4L). Collectively, our results demonstrated that the acetyltransferase and RNA helicase functions are required for breast tumor growth and brain metastasis in vivo.

### NAT10 promotes PHGDH and PSAT1 expression in an RNA helicase domain–dependent manner

**Fig. 5.**
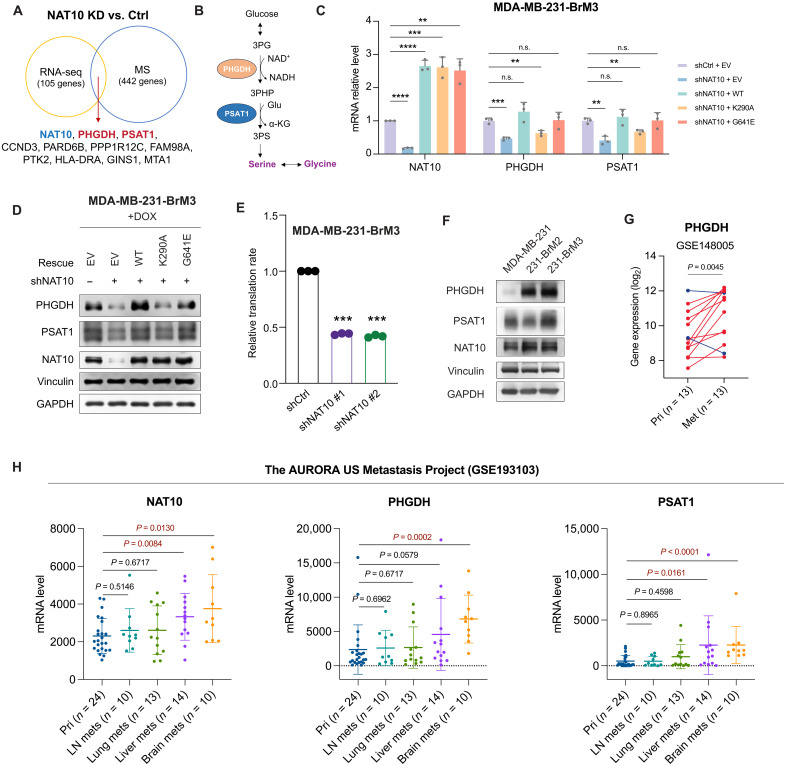
NAT10 promotes PHGDH and PSAT1 expression in an RNA helicase–dependent manner. (**A**) The overlap of differentially expressed genes in RNA-seq and differentially expressed proteins in DIA-MS of 231-BrM3 cells with NAT10 knockdown (shNAT10 #1) versus control cells (shCtrl). For both RNA-seq and DIA-MS, the differentially expressed candidates were defined as *P* < 0.5 and log_2_ (fold change) > 0.3 or <−0.3. CCND3, Cyclin D3; PARD6B, partitioning defective 6 homolog beta; PPP1R12C, protein phosphatase 1 regulatory subunit 12C; FAM98A, family with sequence similarity 98 member A; PTK2, protein tyrosine kinase; HLA-DRA, major histocompatibility complex, class II, DR Alpha; GINS1, GINS complex subunit 1; MTA1, metastasis associated 1. (**B**) Glucose-derived l-serine biosynthesis pathway. Glu, glutamate; NAD^+^, nicotinamide adenine dinucleotide (oxidized form); NADH, reduced form of NAD^+^; α-KG, α-ketoglutarate. (**C** and **D**) Relative mRNA levels (C) and protein levels (D) in 231-BrM3 cells with indicated genetic manipulations. (**E**) The relative translation rate of 231-BrM3 with or without NAT10 knockdown. (**F**) Western blots of indicated proteins in parental MDA-MB-231, 231-BrM2, and 231-BrM3 cell lines. (**G**) *PHGDH* mRNA levels in 13 paired primary and metastatic breast tumors from our previous dataset (GSE148005). In total, 11 of 13 metastatic breast tumors show higher *PHGDH* expression than in primary tumors (lines in red). Met, metastasis. (**H**) *NAT10*, *PHGDH*, and *PSAT1* mRNA levels in primary breast tumors and their matched metastases in the AURORA US Metastasis Project (GSE193103). Pri, primary breast tumors; LN mets, lymph node metastasis. *P* values are marked in red if lower than 0.05. Unpaired Student’s *t* test was used in (C) and (E), while paired Student’s *t* test was used in (G) and (H). **P < 0.01; ***P < 0.001; ****P < 0.0001.

To elucidate the molecular effects and biological pathways of NAT10 depletion, we performed RNA sequencing (RNA-seq) analysis of NAT10-depleted and control 231-BrM3 cells. The most enriched biological process (BP) terms in Gene Ontology (GO) analysis of differentially expressed genes [*P* < 0.05, log_2_(fold change) > 0.3 or <−0.3] included regulation of cell proliferation, cell migration, l-serine biosynthetic process, and cellular response to glucose stimulus (fig. S6A). To narrow down the downstream effector candidates, we also performed data-independent acquisition mass spectrometry (DIA-MS) analysis (*29*) of *NAT10* knockdown and control 231-BrM3 cells. The most enriched BP terms among the differentially expressed proteins (DEPs) included terms related to DNA replication, translation, and l-serine biosynthetic process (fig. S6B). When overlapping differentially expressed candidates from RNA-seq and DIA-MS, we identified 11 potential downstream factors, which showed consistent changes (Fig. 5A).

Among the 10 potential downstream effectors of NAT10, PHGDH and PSAT1 are catalyzing enzymes in the glucose-derived serine/glycine synthesis pathway (Fig. 5B), in which PHGDH oxidizes the glycolytic intermediate 3-phosphoglycerate (3PG) to 3-phosphohydroxypyruvate (3PHP), which is subsequently converted to 3-phosphoserine (3PS) by PSAT1 and eventually to serine by phosphoserine phosphatase. Serine is catabolized to generate glycine by mitochondrial serine hydroxymethyltransferase 2 or cytosolic serine hydroxymethyltransferase 1 (*30*, *31*). Serine and glycine are essential resources for protein synthesis and crucial for cell proliferation (*30*, *31*). PHGDH-mediated l-serine biosynthesis is crucial for cancer cells to survive the serine- and glycine-limited microenvironments (*32*, *33*).

To validate whether NAT10 regulates PHGDH and PSAT1 in 231-BrM3, we turned to *NAT10* knockdown cell lines and confirmed that their mRNA levels significantly decreased when NAT10 was depleted (Fig. 5C). We further interrogated which function of NAT10 is critical for this regulation by using WT NAT10–, K290A mutant–, or G641E mutant–rescued cell line. PHGDH and PSAT1 expression could be rescued by WT NAT10 (Fig. 5C). Their expression could be rescued by G641E mutant as well, but not K290A mutant (Fig. 5C). This RNA helicase–dependent regulation of NAT10 could also be validated at the protein level (Fig. 5D). These data suggest that NAT10 promotes PHGDH and PSAT1 expression in an RNA helicase–dependent manner. Because the enriched GO terms of DEPs in NAT10 KD versus control groups included “cytoplasmic translation” and “translation,” we further measured protein translation rate when NAT10 was depleted and showed that silencing *NAT10* impaired global translation rate (Fig. 5E), consistent with the translational regulation role of NAT10.

### NAT10, PHGDH, and PSAT1 are highly expressed in brain metastases

We examined the expression levels of NAT10 and its downstream factors in parental MDA-MB-231 cell line and its brain metastasis derivatives. 231-BrM3 expressed the highest levels of NAT10, PHGDH, and PSAT1 (Fig. 5F and fig. S6C). Both mRNA and protein levels of PHGDH increased proportionally to the increasing brain metastatic ability of 231-BrM2 and 231-BrM3 (Fig. 5F and fig. S6C). Consistently, RNA-seq results showed up-regulation of *PHGDH* in brain metastasis derivatives, including 231-BrM2 in our previous RNA-seq dataset (GSE138122) (fig. S6D) (*34*) and MDA-MB-231 brain metastasis variant (231-BR) in a publicly available RNA-seq dataset (GSE183862) (fig. S6E) (*35*).

To examine the relevance of our findings in human breast tumors, we analyzed our previously published dataset containing 13 primary breast cancer tissues and their paired metastases (GSE148005) (*8*). *PHGDH* was highly expressed in 11 of 13 metastases compared to the paired primary breast cancer tissues (Fig. 5G). We further investigated the expression of *NAT10*, *PHGDH*, and *PSAT1* in the AURORA US Metastasis Project (GSE193103) (*36*), a larger cohort with paired breast cancer metastases and primary tumors. *NAT10*, *PHGDH*, and *PSAT1* were expressed at higher levels in metastases than in the primary breast tumors (fig. S6F). Among all main metastasis sites of breast cancer, brain metastases exhibited the highest levels of *NAT10*, *PHGDH*, and *PSAT1*, followed by liver metastases (Fig. 5H). Consistent with our findings, several recent studies reported that high expression of *NAT10*, *PHGDH*, or *PSAT1* is correlated with the poor survival in breast cancer, especially the TNBC (*37*–*40*).

### Depletion of PHGDH or PSAT1 sensitizes cancer cells to serine/glycine deprivation

PHGDH and PSAT1 are essential enzymes for the glucose-derived serine/glycine biosynthesis pathway; using glucose, metastatic breast cancer cells synthesize serine/glycine to survive the amino acid–limited brain microenvironments (*31*–*33*). To investigate the phenotypes of metastatic breast cancer cells under nutrient-limited conditions, we made a customized cerebrospinal fluid (CSF)–like medium that contains limited serine/glycine (*33*) to mimic the nutrient-limited brain microenvironment. We first examined the potential impact of PHGDH and PSAT1 expression levels on the intracellular serine level in 231-BrM3 cells under serine-limited conditions by culturing PHGDH- or PSAT1-depleted and control 231-BrM3 cells in serine/glycine-limited CSF-like medium. Consistent with the idea that PHGDH and PSAT1 are de novo serine synthesis enzymes in 231-BrM3 cells, we observed decreased intracellular serine level in PHGDH- or PSAT1-depleted 231-BrM3 cells compared to the control cells using liquid chromatography (LC)–MS (fig. S6, G and H).

**Fig. 6.**
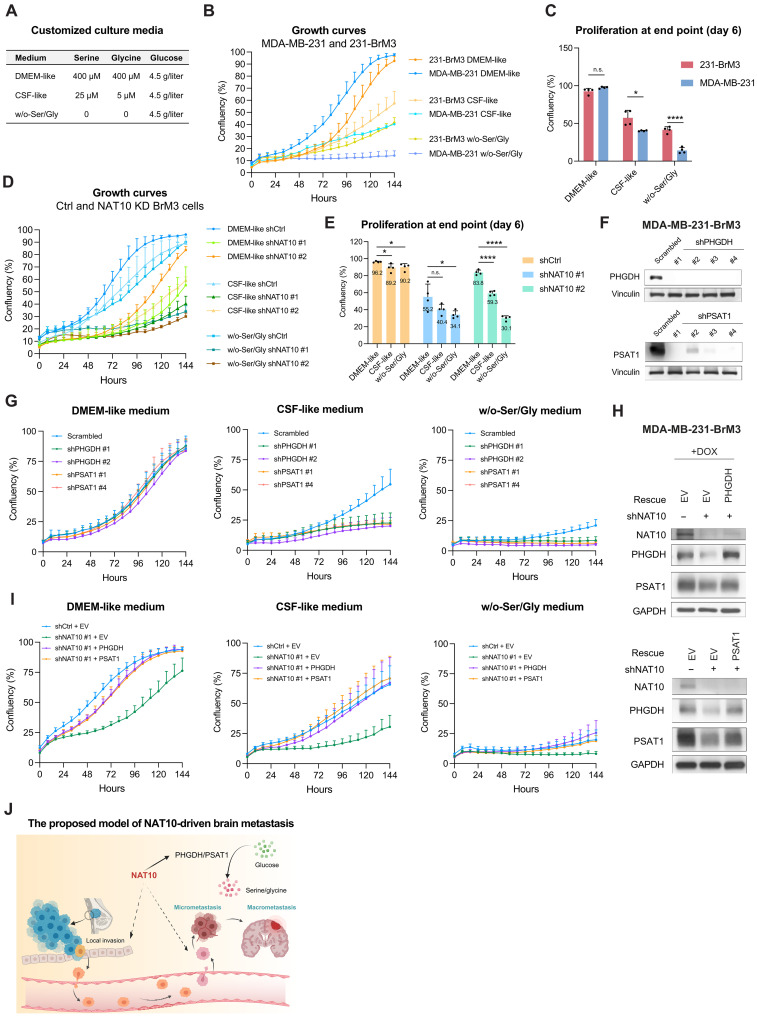
NAT10-regulated PHGDH and PSAT1 are critical for the proliferation of metastatic breast cancer cells under serine/glycine-limited conditions. (**A**) The concentration of serine, glycine, and glucose in DMEM-like, CSF-like, and w/o-Ser/Gly media. (**B** and **C**) Growth curves of 231-BrM3 and MDA-MB-231 cells in customized culture media (B) and the confluency at the end point (day 6) (C). (**D** and **E**) Growth curves of 231-BrM3 cells with or without NAT10 knockdown in customized culture media (D) and the confluency at the end point (day 6) (E), with average confluency labeled. (**F**) Western blots of 231-BrM3 cells with scrambled control and shRNA against *PHGDH* or *PSAT1*. (**G**) Growth curves of control or 231-BrM3 cells with PHGDH or PSAT1 knockdown in customized culture media. (**H**) Validation of PHGDH or PSAT1 reexpression in NAT10-depleted 231-BrM3 cells by Western blots. (**I**) Growth curves of control cells, NAT10-depleted 231-BrM3 cells with EV, PHGDH rescue, or PSAT1 rescue in customized culture media. (**J**) A model depicting the roles of NAT10 in brain metastasis, created with BioRender. Unpaired Student’s *t* test was used in (C) and (E). *P < 0.05; ****P < 0.0001.

In addition to CSF-like medium, we made an even harsher medium, i.e., w/o-Ser/Gly, which does not contain serine/glycine at all, compared to the Dulbecco’s modified Eagle’s medium (DMEM)–like medium that contains high levels of serine/glycine (Fig. 6A). When cultured in DMEM-like medium, 231-BrM3 and parental MDA-MB-231 proliferated similarly, with no significant difference at the end point (day 6) (Fig. 6, B and C). However, 231-BrM3 cells proliferated faster than MDA-MB-231 cells in CSF-like medium or w/o-Ser/Gly medium (Fig. 6, B and C). These results indicated that 231-BrM3 cells, which show much higher expression of PHGDH compared with parental MDA-MB-231 cells (Fig. 5F), proliferate better in a serine/glycine-limited or -depleted environment. This phenotype is likely due to the enhanced de novo serine synthesis ability in 231-BrM3, as indicated by the higher level of intracellular serine level (fig. S6I).

To interrogate whether NAT10 contributes to the better growth of 231-BrM3 cells in serine/glycine-limited or -depleted medium, we cultured 231-BrM3 cells with or without NAT10 depletion in these three customized media and monitored their growth curves. NAT10 depletion led to a more notable difference in proliferation in CSF-like or w/o-Ser/Gly medium than in DMEM-like medium (Fig. 6, D and E).

To further explore the roles of PHGDH and PSAT1 in the proliferation of 231-BrM3 in the brain microenvironment, we depleted them using multiple shRNAs (Fig. 6F) and monitored their proliferation rate in the abovementioned three culture media. As expected, a significant difference in proliferation of these cells was only observed in CSF-like and w/o-Ser/Gly media, but not in DMEM-like medium (Fig. 6G). To address whether NAT10 regulates the proliferation of 231-BrM3 through PHGDH and PSAT1, we reexpressed PHGDH or PSAT1 in NAT10-depleted 231-BrM3 cells (Fig. 6H) and found that either PHGDH or PSAT1 restoration rescued the proliferation in DMEM-like or serine/glycine-limited medium (Fig. 6I). Collectively, these results showed that NAT10 and its downstream factors PHGDH/PSAT1 are crucial for the proliferation of metastatic breast cancer cells in the serine/glycine-limited environment (Fig. 6J).

## DISCUSSION

In the current study, we performed in vivo and in vitro screens of epigenetic dependencies for BCBM and identified NAT10 as a top dropout hit. We established NAT10 as a driver of BCBM through extensive in vitro and in vivo interrogations. In vitro assays showed that the RNA helicase domain, but not the NAT domain, is the most crucial domain for the growth and migration of metastatic breast cancer cells. In vivo data revealed that both RNA helicase and NAT domains are essential for tumor growth and brain metastasis. Furthermore, we demonstrated that NAT10 regulates the expression levels of PHGDH and PSAT1 in an RNA helicase–dependent manner. NAT10 promotes PHGDH and PSAT1 expression to ensure a better survival in a serine/glycine-limited environment, suggesting that NAT10 functions as a master regulator of brain metastasis and participates in multiple steps of metastatic cascade (Fig. 6J). One limitation of our study is that we relied on immunodeficient mice for our in vivo studies. Future investigation using immunocompetent mice will reveal the contributions of the immune system to metastasis regulation by NAT10.

Our rescue experiments confirmed that G641 of NAT10 is a critical residue for its acetyltransferase activity. In addition, we found that the mutation of K290A impaired its ability to acetylate 18*S* rRNA (Fig. 4C), suggesting that the adenosine triphosphatase activity of the RNA helicase is important for NAT10 to perform RNA acetylation. K426 is reported as an autoacetylation site of NAT10, and K426 acetylation is required for NAT10 to activate rRNA transcription (*28*). Here, we uncovered that K426 is crucial for the acetyltransferase activity of NAT10 (Fig. 4C), adding insights into this understudied site. The mechanism by which NAT10 autoacetylation contributes to its acetyltransferase activity requires further study. Our functional studies suggest the RNA acetyltransferase function of NAT10 is less important for breast cancer cell proliferation in vitro (Fig. 4, D to F). Our results are consistent with the previous findings that yeast Kre33 and human NAT10 acetylate 18*S* rRNA and tRNA; however, rRNA acetylation is dispensable for yeast cell growth (*11*). In contrast, we also found that the NAT function of NAT10 is required for breast tumor growth and metastasis in mice. These results indicate that NAT10 N-acetyltransferase inhibitors could be used to treat breast cancer metastasis.

We also show that breast cancer cell growth and NAT10 downstream genes *PHGDH* and *PSAT1* are regulated by NAT10 in an RNA helicase activity–dependent manner. RNA helicases are a large class of enzymes that play pleiotropic functions at multiple steps of the gene expression processes, and the two largest families in humans are DEAD box or DEAH box proteins (*41*). Structurally, the RNA helicase domain of NAT10 shares many conserved characteristics with the DEAD box helicases (*42*, *43*). These RNA helicases engage in several steps of RNA metabolism from splicing, nuclear export, translation, and mRNA decay to mRNA transport and storage. However, these RNA helicases interact with RNA in a sequence-independent manner (*44*). Thus, in addition to further characterizing the detailed mechanisms used by the helicase domain of NAT10, it will also be intriguing to characterize additional domains within NAT10 or protein binding partners of NAT10 that render its substrate specificity in future experiments. Given that NAT10 helicase activity regulates two fundamental catalyzing enzymes of glucose-derived serine/glycine biosynthesis pathway, limited serine and glycine in brain metastasis microenvironment may confer sensitivity to NAT10 helicase targeting agents.

## MATERIALS AND METHODS

### Antibodies and chemicals

Antibodies used include anti–FLAG-M2 (Sigma-Aldrich, F1804, RRID: AB_262044), anti–α-tubulin [Cell Signaling Technology (CST), 3873, RRID: AB_1904178], anti-vinculin (CST, 13901, RRID: AB_2728768; Sigma-Aldrich, v9131, RRID: AB_477629), anti–glyceraldehyde-3-phosphate dehydrogenase (GAPDH; ABclonal, AC001, RRID: AB_2619673), anti-NAT10 (Proteintech, 13365-1-AP, RRID: AB_2148944; Bethyl, A304-385A, RRID: AB_2620580), anti-ac4C (Abcam, ab252215, RRID: AB_2827750), anti–cyclin D1 (CST, 2978, RRID: AB_2259616), anti-V5 (Invitrogen, MA5-15253, RRID: AB_10977225), anti-PHGDH (Proteintech, 14719–1-AP, RRID: AB_2283938), and anti-PSAT1 (Proteintech, 10501-1-AP, RRID: AB_2172597). Chemicals used include nocodazole (Sigma-Aldrich, M1404).

### shRNA library and lentivirus for screen

Frozen bacterial stocks harboring the shRNA library were generated by the Thomas Westbrook Lab at Baylor College of Medicine. pGIPZ plasmid harboring hairpins and barcodes was digested with Xho I and Mlu I and subcloned into the pINDUCER10 plasmid. The list of hairpin sequences is available in table S1. For virus generation, human embryonic kidney (HEK) 293 T cells were transfected with 1.2 μg each of pHDM-VSV-G, pHDM-Tat1b, pRC-CMV-Rev1b and pHDM-Hgpm2 packaging plasmids along with 11.2 μg of lentiviral plasmid. Opti-MEM and TransIT-293 Transfection Reagent (Mirus, MIR2700) were used following the manufacturer’s protocol. Viruses were collected at 48 and 72 hours, filtered through a 0.45-μm filter.

### Establishment of the in vivo selected 231-BrM3

The in vivo selected MDA-MB-231 brain metastasis derivative cell line 231-BrM2 was previously obtained from the Joan Massagué Lab at the Sloan Kettering Institute at Memorial Sloan Kettering Cancer Center. To ensure its capability of colonizing the brain, 1 × 10^5^ 231-BrM2 cells were injected into nude mice intracardially. Brain metastases were monitored weekly with in vivo live imaging. At week 5, the mice were euthanized, and the brain tissue was harvested and minced physically with razor blade and digested with medium containing 0.125% type III collagenase and 0.1% hyaluronidase for 1 hour at 37°C and then with trypsin for another 15 min. After digestion, the tissue fragments were washed twice with 1 × phosphate-buffered saline (PBS) and transferred to a culture dish to sit down. Cells were split for a few passages until the fibroblasts were gone. The in vivo selected cell line, named 231-BrM3, was tested for green fluorescent protein expression with fluorescence-activated cell sorting (FACS) and tested for mycoplasma contamination. Last, 231-BrM3 cells were reinjected into nude mice intracardially in parallel with 231-BrM2 cells to compare the brain colonization ability.

### Cell culture and media

MDA-MB-231, 231-BrM2, 231-BrM3, 231-BoM, 231-LM2, H2030-BrM3, MDA-MB-157, and HEK293T cells were cultured in DMEM supplemented with 10% (v/v) fetal bovine serum (FBS; Gibco) and penicillin/streptomycin (pen/strep). T47D was cultured in RPMI 1640 supplemented with 10% FBS (Gibco) and pen/strep. Cells were periodically tested for mycoplasma contamination and authenticated using short tandem repeat profiling. For the generation of cell lines for screening in 231-BrM3, viruses harboring pINDUCER10 constructs were titrated using the target cell lines, and cells were infected at a multiplicity of infection of 1. 231-BrM3 stable cells were selected with puromycin (0.8 μg/ml).

DMEM-like, CSF-like, and w/o-Ser/Gly culture media were made by using DMEM without glucose, glutamine, serine, glycine, and sodium pyruvate (US Biological, D9802-01), supplemented with 10% (v/v) inactivated dialyzed inactivated FBS (Gibco, 26400044), pen/strep, GlutaMAX Supplement (2 mM; Gibco, 35050061), and d-glucose (4.5 g/liter). DMEM-like medium contains 400 μM serine and 400 μM glycine, CSF-like medium contains 25 μM serine and 5 μM glycine, and w/o-Ser/Gly does not contain serine and glycine. All pH-adjusted complete media were filtered through a 0.22-μm filter before use.

### In vitro and in vivo screens using minipools of cell lines

Minipools were created by equally mixing 11 to 13 individual inducible epigenetic regulator knockdown cell lines with shBUD31 and shCHEK1 as positive and negative controls, respectively. For in vitro screen, minipool cells were plated onto 10-cm dishes with or without DOX (1 μg/ml). A portion of minipool cells were collected as day 0 samples as the controls. Every 2 days, the cells were pelleted, and all samples were collected for gDNA isolation and gDNA qPCR. For in vivo screen, 5 × 10^5^ minipool cells were injected into nude mice through the left ventricle for the brain or bone metastasis screen. Brain or bone metastases were monitored weekly with in vivo live imaging. At the end point (5 weeks), the mice were euthanized, and the target tissues were harvested for gDNA isolation and gDNA qPCR. For the screening readout analyses, all qPCR results were normalized to the value from day 0. The fold change was obtained from ±DOX for both in vitro and in vivo screens.

### Animal studies

Female athymic nude mice (*Foxn1*^nu/nu^, National Cancer Institute stock no. 553) were purchased from Charles River Laboratories for brain metastasis (6-week-old mice) or bone metastasis (5-week-old mice) experiments with human cell lines. For in vivo brain metastasis screen, 5 × 10^5^ 231-BrM3 cells in 0.1 ml of saline were injected intracardially. Mice under the treated group were placed on DOX chow (Envigo, TD.01306) 5 days before injection. All the in vivo metastasis signals, including brain metastases and extracranial metastases, were monitored by weekly bioluminescence imaging. Briefly, anesthetized mice (ketamine 100 mg/kg and xylazine 10 mg/kg) were injected retro-orbitally with d-Luciferin (150 mg/kg) and imaged with an IVIS Spectrum Xenogen machine coupled to Living Image acquisition and analysis software (Perkin Elmer). Luminescence signals were quantified at the indicated time points. Values of luminescence photon flux of each time point were normalized to the value obtained immediately after xenografting (day 0).

For brain colonization validation assays, cell lines were treated with DOX for 3 days before injection, and 0.1 ml of PBS containing 20,000 control shRNA (shCtrl) or shRNA targeting *NAT10* (shNAT10) 231-BrM3 cells or 250,000 H2030-BrM3 was injected into the left ventricle of mice. For bone colonization validation assays, 50,000 shCtrl or shNAT10 231-BoM cells were injected. Mice were placed on DOX chow (Envigo, TD.01306) 5 days before injections. In vivo bioluminescence imaging was performed as described above to monitor brain metastasis, bone metastasis, or metastasis outside of the brain or bone.

For mammary fat pad tumor assays, 1 × 10^6^ control and shNAT10 #1 231-BrM3 cells were resuspended in 0.1 ml of saline and Matrigel (Corning, 356231) mix and then injected into mammary fat pad (the fourth mammary glands) of 6-week-old NOD-SCID mice as described previously (*45*). Tumors were monitored weekly by in vivo bioluminescence imaging as described above. Tumor volume at the end point was calculated by the measurements of tumor length (*L*) and width (*W*) as *V* = *L* × *W*^2^/2. Mice were euthanized when primary tumors reached 1000 mm^3^. This work was performed according to National Institutes of Health guidelines, and all animal procedures were approved under protocol 2121-11286 by the Institutional Animal Care and Use Committee of Yale University.

### Tissue harvest and gDNA isolation

Mice were euthanized, and the whole body was perfused with 10 ml of PBS. For gDNA isolation, the harvested tissue was placed into a microcentrifuge tube and snap frozen with liquid nitrogen. The frozen tissues were then placed into an aluminum block on dry ice. Each tube of the tissue was allowed to thaw enough for further mincing with surgical scissors and then refrozen by dipping them in liquid nitrogen bath. This process was repeated two and three times until no visible tissue chunk was observed. Sixty milligrams of homogenized tissue was then aliquoted out and processed with the DNeasy Blood & Tissue Kits (QIAGEN, 69504) following the manufacturer’s protocols.

### Barcode gDNA qPCR

For barcode qPCR, isolated gDNA was diluted with water and Fast SYBR Green Master Mix (Thermo Fisher Scientific, 4385614), and barcode primers were designed to amplify only one barcode sequence among the 100 unique barcodes in the entire library. The primer set targeting the thyroid response element in pINDUCER10 was used for normalization. The full list of barcode qPCR primers used for the detection of hairpin abundance is available in table S2.

### Histopathology

Mice were euthanized by CO_2_ asphyxiation, and the brain was harvested, immersion fixed in 10% neutral buffered formalin, processed, sectioned, and stained by H&E with routine methods by Yale Research Histology (Department of Pathology). Digital light microscopic images were recorded using a Keyence BZ-X700 immunofluorescent microscope.

### Knockdown and rescue cell lines

For cloning of the WT NAT10 and point mutants, primers flanking with Eco RI were designed against pICE-FLAG-NAT10-siR-WT and pICE-FLAG-NAT10-siR-G641E. Two-step PCR was performed to generate shRNA-resistant mutants. The final PCR products were then cloned into pBABE-hygro plasmid by cut-and-paste method. The K290A and K426R mutants were further generated using site-directed mutagenesis. For cloning of WT NAT10 and K290A into the inducible expression backbone, BP cloning primers were designed against pICE-FLAG-NAT10-siR-WT. The PCR product was then used for BP (Thermo Fisher Scientific, no. 11789020) or LR (Thermo Fisher Scientific, 11791020) reaction into pDONR-211. The list of cloning oligos can be found in table S3.

The recombinant lentivirus was produced by transient transfection in HEK293T cells with packing plasmid (psPAX2) and envelope VSVG plasmid (pMD2.G) together with shRNA clones or overexpression vectors using Lipofectamine 2000 (Thermo Fisher Scientific). Culture media were harvested 42 to 48 hours after transfection, filtered by a syringe filter with 0.45-μm pore size, and then added into the cells to be infected with polybrene (8 μg/ml). The infected cells were selected by specific antibiotics. Similarly, the recombinant retroviruses were produced by transient transfection of Phoenix amphotropic cells with pBABE-hygro expression vectors using Lipofectamine 2000 (Thermo Fisher Scientific).

For PHGDH and PSAT1 rescue vectors, bacteria expressing pDONR-221-PHGDH or pDONR-221-PSAT1 plasmids were obtained from Yale Cancer Center (YCC) Functional Genomics Core. pLIX403-ccdB-Hygro was generated in our laboratory. Plasmids were extracted, and LR reactions (Thermo Fisher Scientific, 11791020) were performed to generate pLIX403-PHGDH-Hygro or pLIX403-PSAT1-Hygro plasmids, the sequences of which were confirmed by whole plasmid sequencing at Quintara Biosciences. For *PHGDH* and *PSAT1* knockdown 231-BrM3 cell lines, bacterial culture harboring pLKO.1 shRNA plasmids was obtained from YCC Functional Genomics Core or Sigma-Aldrich predesigned shRNA. The extracted plasmid was transfected with packing plasmid (psPAX2) and envelope VSVG plasmid (pMD2.G) using FuGENE 6 Transfection Reagent (Promega, E2691). The following lentivirus harvest, infection, and selection steps were similar to the steps mentioned above. shRNA sequences are available in table S4.

### Colony formation assay, WST-1 cell proliferation assay, and grow curve

Colony formation assays were done by seeding single cells in 6- or 12-well plates. The medium was replenished every 3 days with indicated treatments. Colonies were fixed in 4% paraformaldehyde (PFA), followed by 0.5% crystal violet staining for 30 min at room temperature and rinsed with water. Quantification was performed using the ImageJ software plugin ColonyArea (*46*). Statistical significance was determined using unpaired two-tailed Student’s *t* test performed on intensity values from ColonyArea.

For WST-1 cell proliferation assay, cells were seeded at 1000 cells per well in 100 μl into 96-well white plates (Corning, 9154). One to five days after seeding as indicated, the medium was replicated by a 5% solution of WST-1 reagent (Roche, 11644807001) in medium. The plate was incubated for 1 hour at 37°C. The absorbance at 440 nm was measured, as well as the absorbance at 610 nm as a reference. The absorbance at 610 nm was subtracted from the 440-nm reading. The average of the medium-only absorbance was subtracted from all wells. Unpaired two-tailed Student’s *t* test was used to determine significance.

For growth curves, 10,000 cells per well in 1 ml of DMEM were seeded into a 24-well plate (Costar, 3526); 18 hours after seeding (defined as *t* = 0 hours), the media were replaced with the customized media, and at least four pictures were taken for each well every 8 hours for 6 days with the CELLCYTE X system. Confluency was calculated with the default analyzing software of the CELLCYTE X system. For DOX-inducible knockdown cell lines, DOX was added in DMEM to induce knockdown before the seeding and withdrawn in customized media during the proliferation assays.

### BrdU cell proliferation and cell cycle assays

Cell proliferation was assayed using a Cell Proliferation ELISA (enzyme-linked immunosorbent assay) BrdU kit (Roche, 11647229001). Briefly, 3 × 10^3^ cells were seeded on a 96-well plate, and BrdU was added to the culture medium at 18 hours after plating. BrdU labeling was continued for 1 hour before cell harvest. Absorbance at 450 nm was measured, as well as the absorbance at 690 nm as a reference. Unpaired two-tailed Student’s *t* test was used to determine significance.

For cell cycle analysis, cells were treated with DOX for 3 days to induce shRNA expression and then treated with nocodazole (40 ng/ml) in a serum-free medium for 24 hours for cell synchronization. Cells were then switched back to normal media. At 0-, 9-, 12-, 15-, 18-, and 24-hour time points, cells were collected and fixed in 70% ice-cold ethanol at 4°C overnight. Fixed cells were resuspended in PBS containing 4′,6-diamidino-2-phenylindole (DAPI) (1 μg/ml), and the analysis was performed on a FACS Stratedigm STD-13 cytometer.

### 3D organoid growth assay

Cells were resuspended in a medium containing 2% FBS and 5% growth factor–reduced Matrigel. Cells were then seeded into an ultralow attachment 24-well plate (Corning, 3473) at 1000 cells per well. Every 3 days, 50 μl of Matrigel-containing medium was added to each well to refresh the cells. The spheroid growth was measured with the Promega dual-luciferase reporter assay system (E1910). Briefly, on day 12, spheroids were collected, pelleted, and lysed with 1× passive lysis buffer. Cell lysates were stored at −80°C at least overnight. On the day of luciferase assay, cell lysates were thawed and luciferase assay substrate was added and then the luciferase activity was measured immediately with a plate reader.

### Transwell migration assays

For transwell migration assay, cell culture inserts (Corning, 353097) were placed in 24-well plates. A total of 2 × 10^4^ cells resuspended in medium containing 0.1% FBS were plated onto the upper chamber, and the medium containing 10% FBS was added to the lower chamber. Cells were incubated at 37°C for 5 or 18 hours. At the end point of incubation, cells that had migrated onto the lower membrane surface were fixed with 4% PFA in PBS, stained with DAPI, and counted.

### Apoptosis assays

Cells were treated with DOX for 3 days to induce shRNA expression before performing apoptosis assays. An additional plate of cells was treated with 50 μM etoposide for 48 hours to induce apoptosis as a positive control. On the day of analysis, cells were harvested and stained with annexin V–biotin (BioLegend, 640904), followed by steptavidin-Cy7 (BioLegend, 405208) and Helix NP Blue (BioLegend, 425305). Flow cytometry was performed to record the signals using allophycocyanin and Pacific Blue channels. Analyses were done using FlowJo software.

### Western blot

Cells were lysed in 1× high-salt lysis buffer [50 mM tris (pH 8), 320 mM NaCl, 0.1 mM EDTA, 0.5% NP-40, and 10% glycerol] supplemented with 1× cOmplete protease inhibitor (Roche, 11836153001) and 1× PhosSTOP phosphatase inhibitor (Roche, 4906845001). Cell lysates were vortexed and centrifuged, and the supernatants were subjected to protein quantification by Bradford reagent (Bio-Rad, 5000006) and sample preparation by sample buffer [10% glycerol, 50 mM tris-HCl (pH 6.8), 2% SDS, 0.01% bromophenol blue, and 8% β-mercaptoethanol]. Protein samples were resolved by SDS–polyacrylamide gel electrophoresis according to standard protocol and transferred onto 0.45-μm nitrocellulose membranes (Bio-Rad, 1620115). The membranes were blocked with 1 to 5% milk in TBST (tris-buffered saline with 0.1% Tween 20) at room temperature for at least 10 min. The membranes were incubated with primary antibody diluted under blocking conditions overnight at 4°C. The nitrocellulose membranes were rinsed with 0.1% TBST three times for 5 min and incubated with indicated horseradish peroxidase–conjugated secondary antibody at room temperature for 1 hour. Blots were rinsed three times with 0.1% TBST and visualized using the KwikQuant system (Kindle Biosciences, D1001).

### Reverse transcription qPCR

Total RNA from cells was extracted using the RNeasy Plus Mini Kit (QIAGEN, 74136) protocol. RNA was reverse transcribed into cDNA using the High Capacity cDNA Reverse Transcription kit (Thermo Fisher Scientific, 4385614). The resulting cDNA was diluted with diethyl pyrocarbonate water, and Fast SYBR Green Master Mix (Thermo Fisher Scientific, 4385614) was used for real-time PCR. GAPDH was used for normalization. Samples were run in quadruplicate, and experiments were performed at least three times. Primer sequences are listed in table S5. Unpaired two-tailed Student’s *t* test was used to determine significance.

### RNA sequencing

231-BrM3 cells from knockdown control or shNAT10 group were harvested with QIAzol Lysis Reagent (QIAGEN) and homogenized using QIAshredder tubes (QIAGEN). For each cell line, shRNA sequence was induced with DOX (1 μg/ml) for 3 days, and three biological replicates were harvested at different passages. RNA isolation was performed using miRNeasy with on-column DNase digestion. The External RNA Controls Consortium (ERCC) spike-in RNA was added in proportion to the number of cells obtained during cell counts. Library generation was performed using TruSeq Stranded mRNA library preparation kit (Illumina). Paired-end sequencing was performed using an Illumina HiSeq 4000 sequencer, generating an average of 59 million reads per library. Reads were aligned to hg38, and gene counts to GENCODEv96 transcripts were obtained using STAR aligner v2.7.0 with default parameters. The hg38 and GENCODEv96 annotations were appended to include the ERCC sequences. DESeq2 was used to obtain differential gene expression, and HTSFilter was used to filter for expressed genes. Significant differences were identified using a Benjamini-Hochberg–adjusted *P* value cutoff of 0.05. The differentially expressed gene list is available in table S6. The GO analysis of differentially expressed genes was performed with the Database for Annotation, Visualization and Integrated Discovery (DAVID; https://david.ncifcrf.gov/) (*47*) based on Knowledgebase v2024q1.

### Data-independent acquisition mass spectrometry

shCtrl and NAT10 knockdown (shNAT10 #1) 231-BrM3 cells were induced with DOX (1 μg/ml) for 3 days, and three biological replicates were harvested at different passages. The protein extraction, digestion, DIA-MS, and data analysis were described in Mehnert *et al.* (*29*). The protein list of DIA-MS is available in table S7. The GO analysis of DEPs was performed with DAVID (https://david.ncifcrf.gov/) based on Knowledgebase v2024q1.

### Serine quantification using LC-MS

A total of 5 × 10^5^ to 8 × 10^5^ cells per well were seeded onto six-well dishes for 24 hours before harvesting. For each group, cells on two wells were counted, and the cell numbers were used for normalization, while cells on the other four wells were harvested for metabolite detection. To prepare cellular metabolite extracts, the cells were washed once with 1 ml of ice-cold PBS and extracted with acetonitrile-methanol-water [27:9:1 (v/v/v)]. After vortexing and centrifugation at 19,000*g* for 20 min at 4°C, supernatants were transferred into LC-MS glass vials for analysis. LC-MS–based analyses were performed on a Q Exactive plus benchtop orbitrap mass spectrometer equipped with an Ion Max source and a HESI II probe, coupled to a Vanquish UHPLC using XBridge BEH Amide XP HILIC Column (2.5 μm, 2.1 × 100 mm; Waters, 186006091). The column oven temperature was 27°C, and the autosampler was set at 4°C. Mobile phase A: 5% acetonitrile, 20 mM ammonium acetate/ammonium hydroxide, and pH 9, Mobile phase B: 100% acetonitrile. LC gradient conditions at a flow rate of 0.220 ml/min as follows: 0 min: 85% B, 0.5 min: 85% B, 9 min: 35% B, 11 min: 2% B, 13.5 min: 85% B, and 20 min: 85% B. The mass data were acquired in the polarity switching, full scan mode in a range of 70 to 1000 *m*/*z* (mass/charge ratio), with resolution at 70,000, AGC target at 3 × 10^6^, maximum injection time at 80 ms, sheath gas flow at 50 U, auxiliary gas flow at 10 U, sweep gas flow at 2 U, spray voltage at 2.5 kV(−) and 3.8 kV(+), capillary temperature at 310°C, and auxiliary gas heater temperature at 370°C. Compound discoverer (Thermo Fisher Scientific) was used for peak picking and intensity.

### Immuno–Northern blot

Equal amounts of total RNA (15 μg) or poly(A) RNA were mixed with four times volume of RNA sample buffer [65% formamide, 22% formalin, 13% 10× Mops, and ethidium bromide (8 μg/ml)] and one time volume of loading dye (50% glycerol, 1 mM EDTA, 0.3% each bromophenol blue, and 0.3% xylene cyanol), heated to 65°C for 15 min, and separated on 1% agarose denaturing gel. Loading control was verified by ultraviolet (UV) imaging before transfer. RNA was transferred onto Amersham Hybond-N+ membranes (GE Healthcare) by capillary transfer using 1× transfer buffer (3 M NaCl, and 0.01 M NaOH) for 1.5 hours. Membranes were rinsed with 6× SSC buffer, cross-linked twice with 150 mJ/cm^2^ in the UV254nm Stratalinker 2400, blocked with 5% nonfat milk in 0.1% PBST for 30 min at room temperature, and probed overnight with anti-ac4C antibody in 1% nonfat milk (1:10,000) at 4°C. Membranes were next washed three times with 0.1% PBST, incubated with horseradish peroxidase–conjugated secondary anti-rabbit immunoglobulin G in 1% nonfat milk at 4°C overnight, washed three times with 0.1% PBST, and developed with the SuperSignal ELISA Femto Maximum Sensitivity Substrate (Thermo Fisher Scientific). Chemiluminescence was detected using KwikQuant Imager camera.

### Translation rate assay

Cells were starved of l-methionine for 30 min and subsequently incubated with 50 μM homopropargylglycine (HPG; Life Technologies, C10186) for 1 to 4 hours in treatment media. Cells were then trypsinized and fixed in 4% PFA. A Click-iT kit (Life Technologies, C10269) was used to label HPG. Labeled cells were analyzed using a CytoFLEX flow cytometer. Translation rates were determined on the basis of the slope of HPG incorporation over time.

### Statistical analysis

Comparisons between two groups were performed using an unpaired two-sided Student’s *t* test unless indicated otherwise. Graphs represent either group mean values ± SEM or individual values (as indicated in the figure legends). For animal experiments, each tumor graft was an independent sample. All experiments were reproduced at least three times.
