# Supplementary material for: An in vivo screen identifies NAT10 as a master regulator of brain metastasis
Source: Sci Adv. 2025 Mar 26;11(13):eads6021. doi: 10.1126/sciadv.ads6021 (PMC11939035; doi:10.1126/sciadv.ads6021)
Supplement: Supplementary file 1 — Figs. S1 to S6 Legends for tables S1 to S7 [file sciadv.ads6021_sm.pdf]

Science Advances
AAAS

Supplementary Materials for

An in vivo screen identifies NAT10 as a master regulator of brain metastasis

Jocelyn F. Chen *et al.*

Corresponding author: Qin Yan, qin.yan@yale.edu

Sci. Adv. **11**, eads6021 (2025)
DOI: 10.1126/sciadv.ads6021

The PDF file includes:

Figs. S1 to S6
Legends for tables S1 to S7

Other Supplementary Material for this manuscript includes the following:

Tables S1 to S7

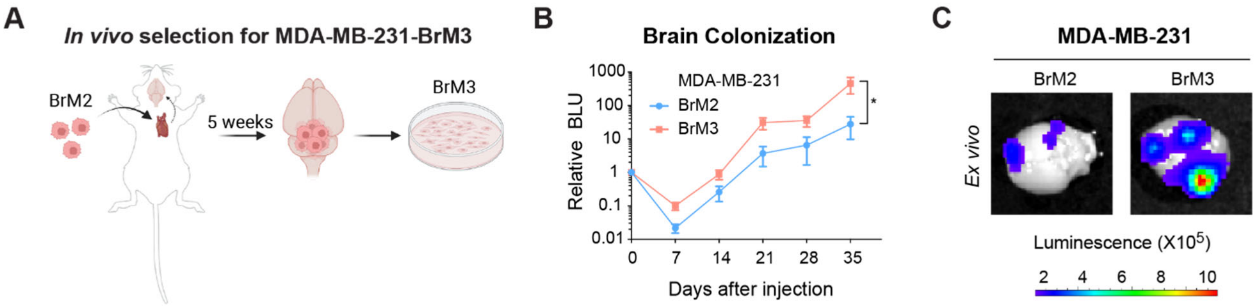

fig. S1. Generation of triple-negative breast cancer cell line with brain organotropism. (**A**) Schematic of *in vivo* selection process to generate MDA-MB-231-BrM3 (231-BrM3) cell line. (**B**) Normalized bioluminescence signals of brain metastases of mice injected intracardiacally with MDA-MB-231-BrM2 (231-BrM2) or 231-BrM3 cells from (**A**). The data represent average ± SEM. Significance was determined using two-tailed student's t-test. *, $P < 0.05$. (**C**) Representative bioluminescence images of mice brain metastases in (A) at day 35.

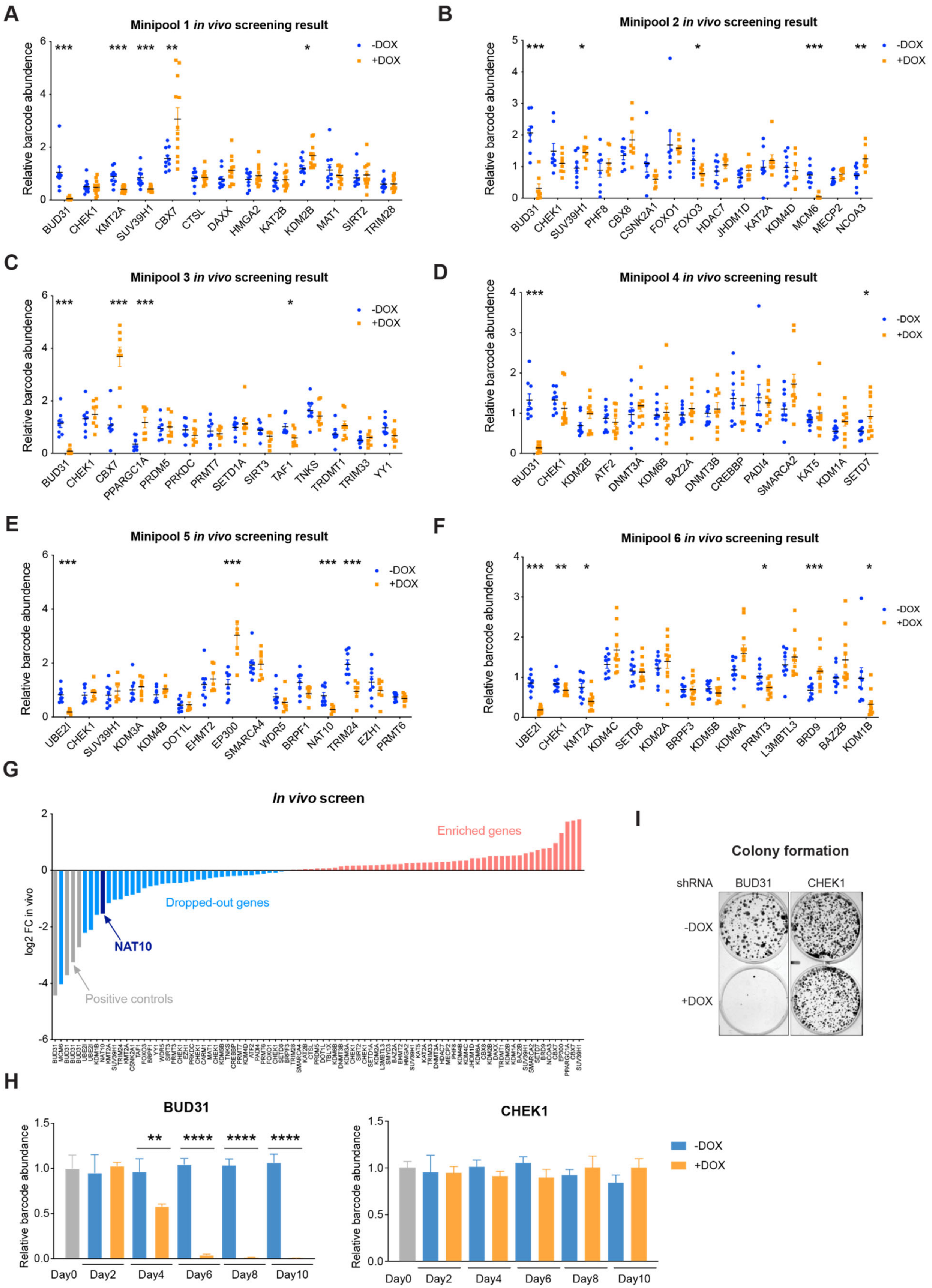
A
Minipool 1 in vivo screening result
Relative barcode abundence
-DOX
+DOX
*** *** *** ** *
BUD31
CHEK1
KMT2A
SUV39H1
CBX7
CTSL
DAXX
HMGA2
KAT2B
KDM2B
MAT1
SIRT2
TRIM28
B
Minipool 2 in vivo screening result
Relative barcode abundence
-DOX
+DOX
*** * * *** **
BUD31
CHEK1
SUV39H1
PHF8
CBX8
CSNK2A1
FOXO1
FOXO3
HDAC7
JHDM1D
KAT2A
KDM4D
MCM6
MECP2
NCOA3
C
Minipool 3 in vivo screening result
Relative barcode abundence
-DOX
+DOX
*** *** *** *
BUD31
CHEK1
CBX7
PPARGC1A
PRDM5
PRKDC
PRMT7
SETD1A
SIRT3
TAF1
TNKS
TRDMT1
TRIM33
YY1
D
Minipool 4 in vivo screening result
Relative barcode abundence
-DOX
+DOX
*** *
BUD31
CHEK1
KDM2B
ATF2
DNMT3A
KDM6B
BAZ2A
DNMT3B
CREBBP
PADI4
SMARCA2
KAT5
KDM1A
SETD7
E
Minipool 5 in vivo screening result
Relative barcode abundence
-DOX
+DOX
*** *** *** ***
UBE2I
CHEK1
SUV39H1
KDM3A
KDM4B
DOT1L
EHMT2
EP300
SMARCA4
WDR5
BRPF1
NAT10
TRIM24
EZH1
PRMT6
F
Minipool 6 in vivo screening result
Relative barcode abundence
-DOX
+DOX
*** ** * * *** *
UBE2I
CHEK1
KMT2A
KDM4C
SETD8
KDM2A
BRPF3
KDM5B
KDM6A
PRMT3
L3MBTL3
BRD9
BAZ2B
KDM1B
G
In vivo screen
log2 FC in vivo
Enriched genes
Dropped-out genes
NAT10
Positive controls
H
BUD31
Relative barcode abundance
** **** **** ****
Day0
Day2
Day4
Day6
Day8
Day10
CHEK1
Relative barcode abundance
Day0
Day2
Day4
Day6
Day8
Day10
-DOX
+DOX
I
Colony formation
shRNA
BUD31
CHEK1
-DOX
+DOX

fig. S2. Screening results of breast cancer brain metastasis. (**A** to **F**) Relative abundance of barcode for shRNA against each epigenetic factor in the library in brain tissues from control and doxycycline-treated mice. (**G**) Waterfall plot of the *in vivo* screening results. Log_2 (fold change) of each cell line was listed in ascending. (**H**) Relative abundance of 231-BrM3 cells stably expressing shRNA against *BUD31* and *CHEK1* after the indicated days of *in vitro* culture under control or doxycycline (1 μg/mL) treatment. Data normalized to abundance at the time of mini-pool mixture (Day 0). (**I**) Colony formation assays of 231-BrM3 cells after 9 days of either control or doxycycline (1 μg/mL) treatment. Significance was determined in (**A** to **F**) and (**H**) using unpaired student's t-test. *, $P < 0.05$; **, $P < 0.01$; ***, $P < 0.001$; ****, $P < 0.0001$.

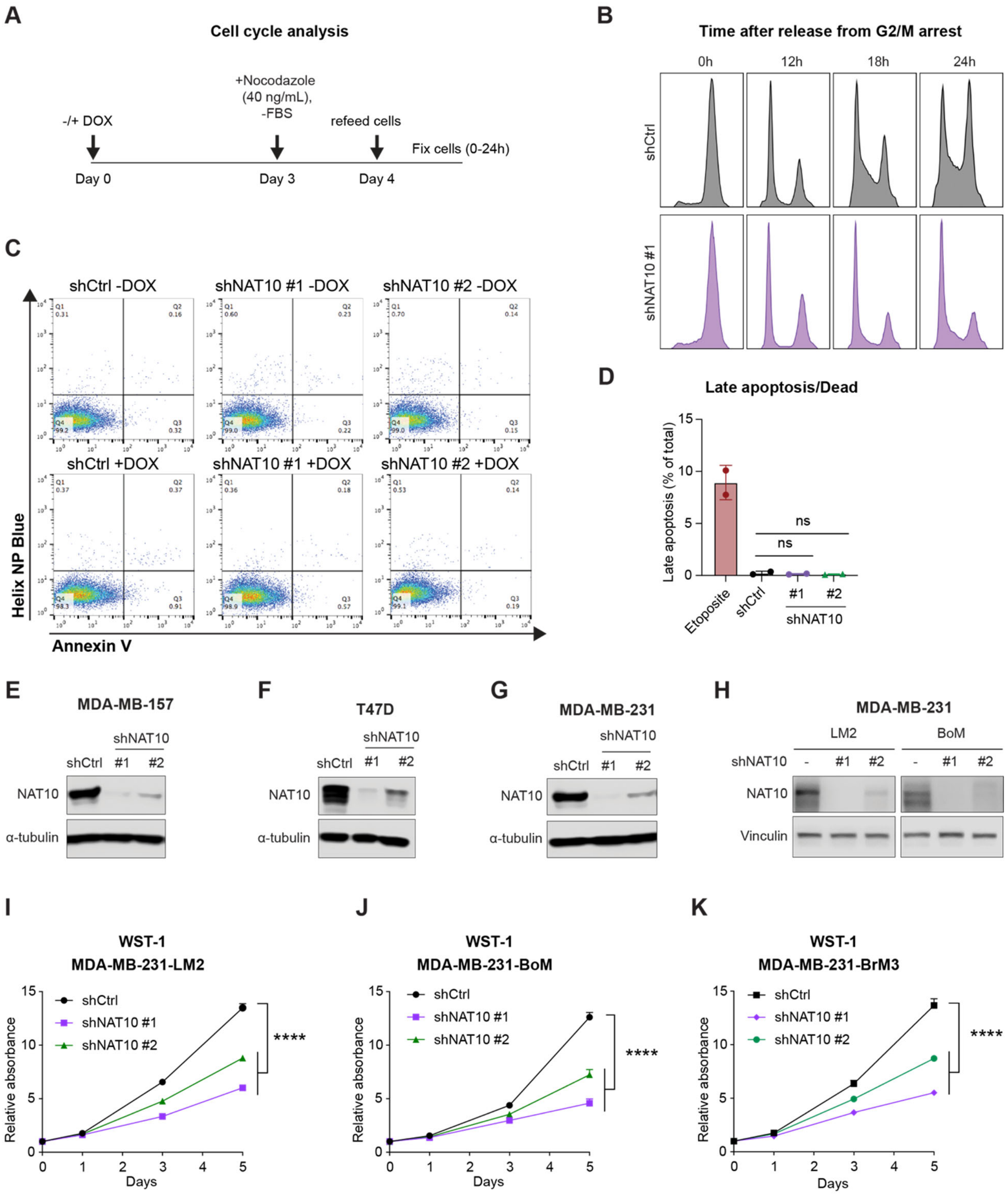

fig. S3. NAT10 depletion disrupts cell cycle and attenuates proliferation of breast cancer cells with distinct metastatic organotropisms. (**A**) Schematic of cell cycle analysis using the synchronizing method of Nocodazole (40 ng/mL) in combination with serum starvation for 24 hours. (**B**) Time after release from G2/M arrest in 231-BrM3 cells. (**C**) Apoptosis assay determined

by Helix NP-Blue and Annexin V staining. 231-BrM3 cells from (**B**) were cultured with regular media with control or doxycycline (1 μg/mL) induction for 3 days. Representative experiment was shown; (**D**) Late apoptosis versus dead cell ratio treated with etoposide and doxycycline. (**E** to **H**) Western blots of NAT10, α-tubulin or Vinculin in indicated cell lines harboring inducible control or NAT10 targeting shRNAs (shNAT10 #1 and shNAT10 #2) after 3 days of doxycycline (1 μg/mL) induction. (**I** to **K**) WST-1 proliferation assays of MDA-MB-231 organotropic derivatives after indicated days of doxycycline (1 μg/mL) treatment. Significance in (**D,** and **I** to **K**) was determined using unpaired student's t-test. ns, not significant; ****, $P < 0.0001$.

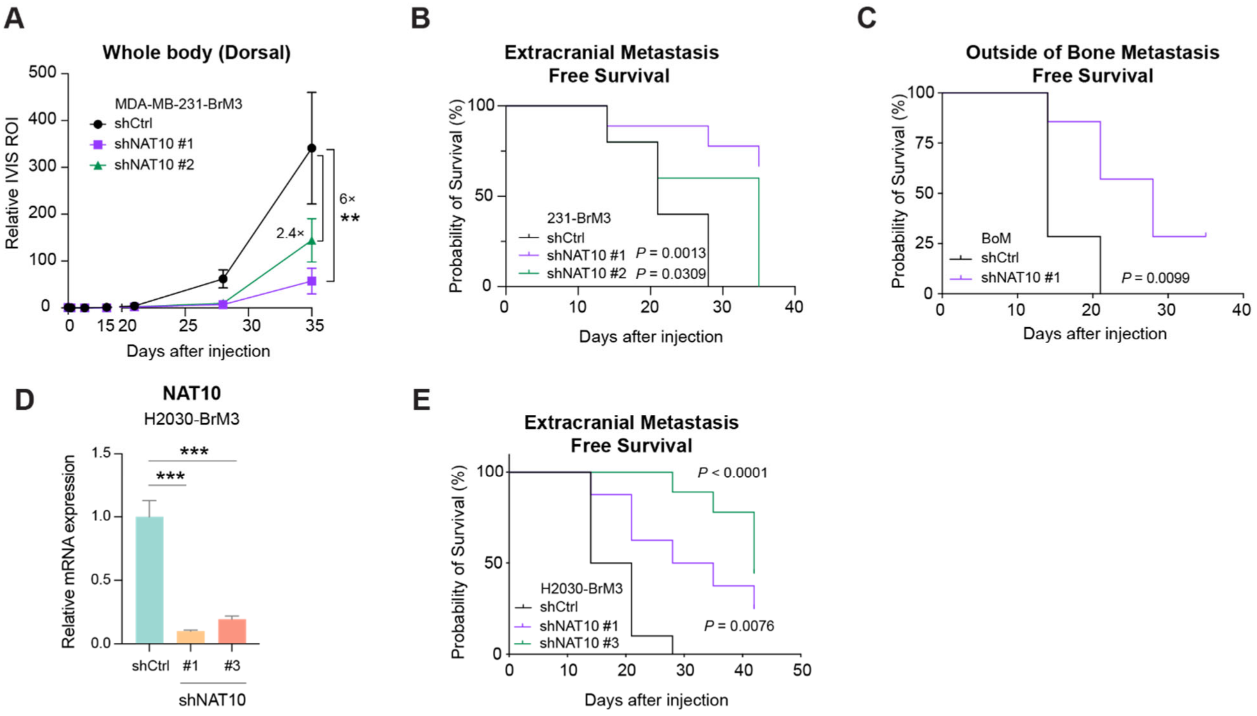

fig. S4. NAT10 is an essential factor in multiple metastasis settings. (**A**) Normalized bioluminescence signals of whole-body metastasis of mice injected intracardially with 231-BrM3 cells harboring inducible control, shNAT10 #1, or shNAT10 #2 and kept under doxycycline chow. The data represent average ± SEM (Unpaired student's t-test; **, $P < 0.01$). (**B**) Kaplan-Meier plot of extracranial metastasis-free survival for mice described in **Fig. 3E**. shCtrl (n=10), shNAT10 #1 (n=9), and shNAT10 #2 (n=10) were analyzed (Log rank Mantel-Cox test). (**C**) Kaplan-Meier plot of metastasis-free survival outside of bone for mice described in **Fig. 3K** (Log rank Mantel-Cox test). (**D**) RT-qPCR analysis of *NAT10* showing the knockdown effects of shRNA targeting NAT10 in H2030-BrM3 cells. (**E**) Kaplan-Meier plot of whole-body metastasis-free survival in **Fig. 3O** (Log rank Mantel-Cox test).

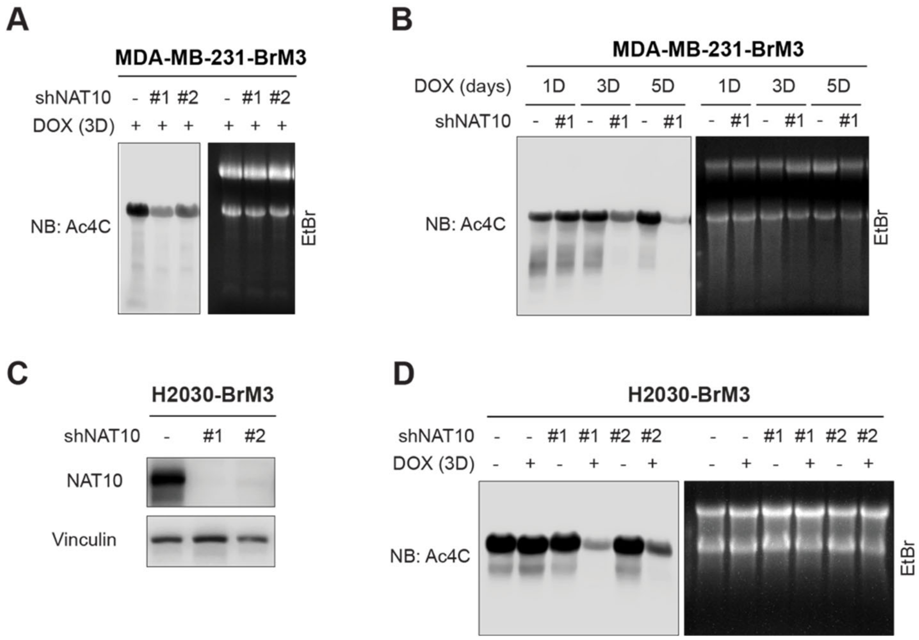

fig. S5. N-acetyltransferase function of NAT10 is active in brain metastatic derivatives. (**A**) Immuno-Northern blots of ac4C modification in total RNA extracted from 231-BrM3 cells harboring inducible control or NAT10-targeting shRNAs after 3 days of doxycycline (1 μg/mL) induction. (**B**) Immuno-Northern blots of ac4C modification in total RNA extracted from 231-BrM3 cells harboring inducible control or NAT10-targeting shRNA after doxycycline (1 μg/mL) induction for 1, 3, or 5 consecutive days. (**C**) Western blot analysis of NAT10 and vinculin showing the knockdown effects of shRNA targeting NAT10 in H2030-BrM3 cells. (**D**) Immuno-Northern blots of ac4C modification in total RNA extracted from H2030-BrM3 cells harboring inducible control or NAT10-targeting shRNAs after 3 days of control or doxycycline (1 μg/mL) induction. In (**A**, **B,** and **D**), denatured agarose gels were stained with EtBr as loading control on the right, in which the upper and lower band were 28S and 18S rRNA, respectively.

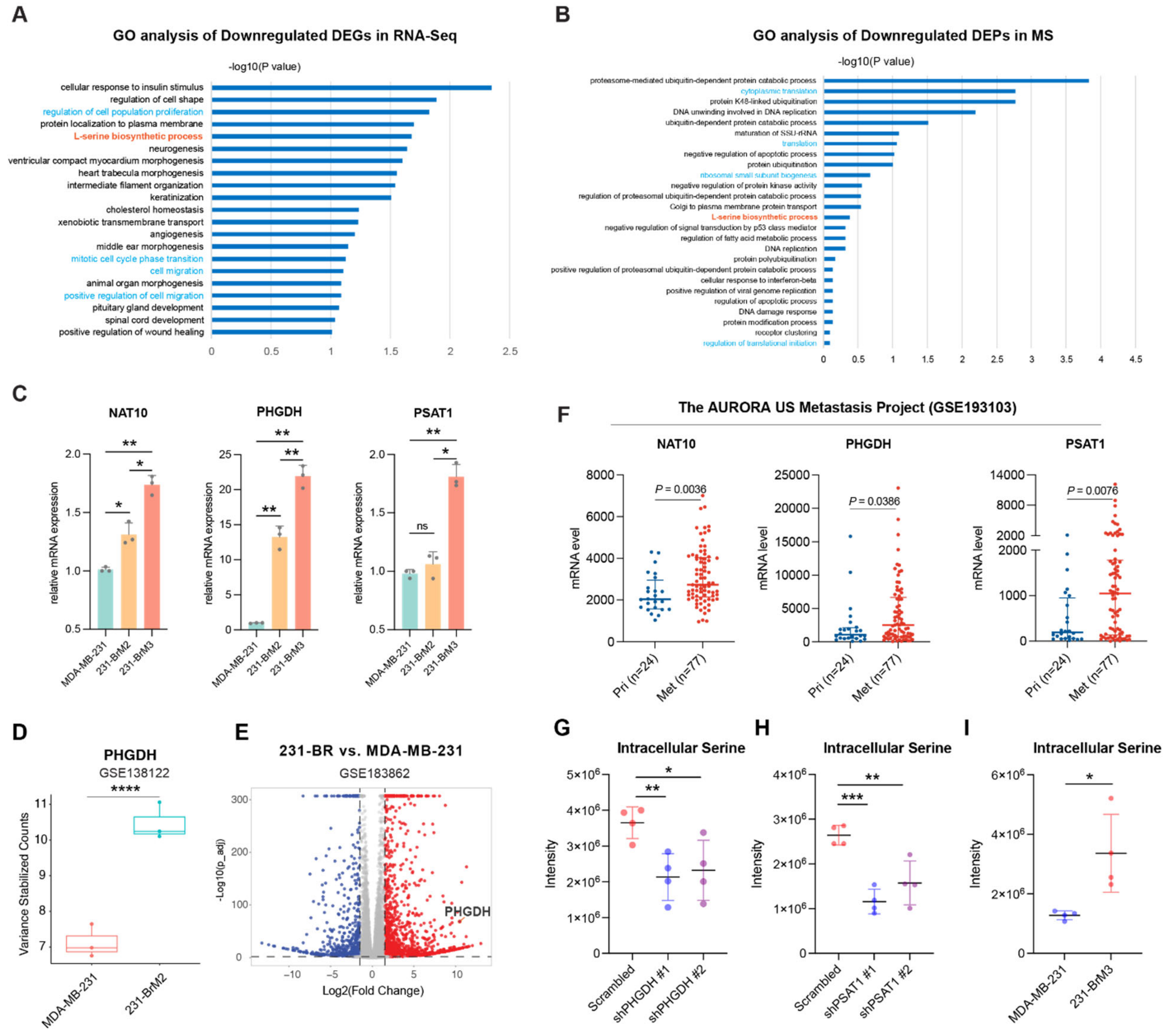

fig. S6. NAT10 and its targets PHGDH and PSAT1 are highly expressed in metastatic breast cancer cells. (**A** and **B**) The enriched Biological Process (BP) terms of differentially expressed genes/proteins in RNA-Seq (**A**)/DIA-MS (**B**) of NAT10 knockdown (shNAT10 #1) versus control (shCtrl) 231-BrM3 cells. For both RNA-Seq and DIA-MS, the differentially expressed candidates were defined as $P < 0.5$ and $\log_2$ (Fold Change) > 0.3 or < -0.3. Terms marked in red indicate the conventional biological processed that NAT10 involves, while terms marked in orange indicate the glucose-derived serine/glycine biosynthesis pathway. (**C**) Relative mRNA levels of NAT10, PHGDH, and PSAT1 in parental MDA-MB-231, 231-BrM2, and 231-BrM3 cell lines. (**D**) mRNA level (normalized counts) of PHGDH in parental MDA-MB-231 and 231-BrM2 from our previously published dataset (GSE138122). (**E**) Volcano plot of the differentially expressed genes in MDA-MB-231 brain metastasis variant (231-BR) versus MDA-MB-231 from a publicly available RNA-seq dataset (GSE183862). (**F**) NAT10, PHGDH, and PSAT1 mRNA levels in primary breast tumors and their matched metastases in The AURORA US Metastasis Project

(GSE193103). Met, metastases. Unpaired student's t-test was used in (**C** and **D**), while paired student's t-test was used in (**F**). *, $P < 0.05$; **, $P < 0.01$; ***, $P < 0.001$. (**G** to **I**) The intracellular serine level of indicated cells when cultured in CSF-Like medium. Unpaired Student-t test; ns, not significant; *, $P < 0.05$; **, $P < 0.01$; ***, $P < 0.001$.

table S1-7. (separate file)

table S1. The list of hairpin sequences for screens.
table S2. The full list of barcode qPCR primers for hairpin abundance detection.
table S3. The list of cloning oligos.
table S4. The sequences of shRNAs used in functional characterization.
table S5. The list of qPCR primers.
table S6. Differentially expressed genes in 231-BrM3 cells with shNAT10 #1 versus 231-BrM3 cells with shCtrl.
table S7. Differentially expressed proteins in 231-BrM3 cells with shNAT10 #1 versus 231-BrM3 cells with shCtrl.
